# Higher Serum Omega-3 Polyunsaturated Fatty Acid Content Is Associated with Improved Long-Term Cardiovascular and Renal Outcomes in Patients with Chronic Kidney Disease

**DOI:** 10.3390/nu18111760

**Published:** 2026-05-30

**Authors:** Małgorzata Sikorska-Wiśniewska, Adriana Mika, Tomasz Śledziński, Izabella Kuźmiuk-Glembin, Alicja Dębska-Ślizień, Michał Chmielewski

**Affiliations:** 1 Department of Nephrology, Transplantology and Internal Medicine, Faculty of Medicine, Medical University of Gdansk, 80-210 Gdansk, Poland; 2 Department of Pharmaceutical Biochemistry, Medical University of Gdansk, 80-210 Gdansk, Polandtsledz@gumed.edu.pl (T.Ś.); 3 Department of Environmental Analytics, Faculty of Chemistry, University of Gdansk, 80-308 Gdansk, Poland; 4 Department of Rheumatology, Clinical Immunology, Geriatrics and Internal Medicine, Medical University of Gdansk, 80-210 Gdansk, Poland

**Keywords:** chronic kidney disease, fatty acid profile, *n*-3 polyunsaturated fatty acids

## Abstract

**Background**: Patients with chronic kidney disease (CKD) exhibit reduced serum levels of *n*-3 polyunsaturated fatty acids (PUFA). Alterations in fatty acid profiles may contribute to increased cardiovascular risk and potentially accelerate CKD progression. **Aim**: The aim of the study was to assess whether fatty acid profiles measured a decade earlier predicted CKD progression and cardiovascular events. Additionally, the impact of dietary patterns at baseline was evaluated. **Methods**: The study comprised 77 patients with CKD whose serum fatty acid profiles had been assessed approximately a decade earlier. Follow-up data on CKD progression, cardiovascular events, and all-cause mortality were collected. Dietary habits were assessed using a food frequency questionnaire. The composite endpoint was defined as renal replacement therapy initiation or occurrence of stroke, myocardial infarction, or death. **Results**: Higher *n*-3 PUFA content was significantly associated with a lower risk of the composite endpoint in Cox regression analysis (HR = 0.63; 95% CI: 0.40–0.99; *p* = 0.044). Significant differences in event-free survival were observed in patients with higher *n*-3/*n*-6 PUFA ratios (log-rank test, χ^2^ = 4.58, *p* = 0.032). Patients who experienced stroke or myocardial infarction had significantly higher levels of *n*-6 PUFA (32.85% vs. 29.94% Mann–Whitney U test, *p* = 0.037) and lower *n*-3/*n*-6 PUFA ratios (0.07 vs. 0.08, Mann–Whitney U test, *p* = 0.045). Eicosapentaenoic acid (EPA) content was significantly lower at baseline in patients who required renal replacement therapy during follow-up compared with those who did not experience this outcome (0.66% [0.48–0.82] vs. 0.88% [0.64–1.13], Mann–Whitney U test, *p* = 0.02). **Conclusions**: Lower serum *n*-3 PUFA levels were observed in patients who reached the composite endpoint during follow-up. A higher *n*-3/*n*-6 PUFA ratio showed a protective effect in survival analysis, and higher EPA content was associated with a lower risk of renal replacement therapy initiation. A more favorable fatty acid profile may be linked to improved cardiovascular and renal outcomes. Further studies are needed to clarify the role of fatty acid profiles in long-term outcomes among patients with CKD, in whom cardiovascular disease remains the leading cause of death.

## 1. Introduction

Cardiovascular disease (CVD) is the leading cause of death among patients with chronic kidney disease (CKD). Given the continuously increasing prevalence of CKD, the identification of risk factors contributing to adverse outcomes in this population remains of particular importance. The clinical phenotype of CVD in patients with CKD differs from that observed in the general population, as non-atherosclerotic factors—including vascular calcification, valvular dysfunction, electrolyte disturbances, and fluid overload—play a significant role. Traditional cardiovascular risk factors include hypertension, diabetes, smoking, obesity, and hyperlipidemia. In CKD population, the association between hyperlipidemia and cardiovascular outcomes is weaker than in the general population, and lipid-lowering therapies appear to be less effective. Notably, in end-stage CKD, low cholesterol levels have been associated with worse prognosis, a phenomenon referred to as “reverse epidemiology” [1]. Non-traditional CVD risk factors in CKD also include anemia, proteinuria, chronic inflammation, oxidative stress, and potentially disturbances in serum fatty acid profiles. Beyond conventional lipid fractions, CKD-related dyslipidemia involves alterations in fatty acid composition, as shown in our studies, including reduced levels of beneficial polyunsaturated fatty acids (PUFAs) [2,3]. Fatty acids lacking double bonds are classified as saturated fatty acids (SFAs), whereas monounsaturated fatty acids (MUFAs) contain a single double bond, while PUFAs contain multiple double bonds. Among PUFAs, the two principal families are *n*-3 and *n*-6 fatty acids. Diets rich in *n*-3PUFA which main representatives are eicosapentaenoic acid (EPA) and docosahexaenoic acid (DHA), found in fish oil, are considered cardioprotective. Evidence regarding the effects of *n*-6 PUFA intake and circulating concentrations remains inconsistent. Some studies suggest that high levels of *n*-6 PUFAs—whose intake is particularly elevated in Western dietary patterns—may be associated with an increased risk of CVD [4]. Other reports indicate an inverse association between total *n*-6 PUFA concentrations and CVD risk [5]. Shoji et al. showed that *n*-3/*n*-6 PUFA ratios were associated inversely with CVD events in hemodialysis population [6]. PUFAs that are incorporated into cell membrane phospholipids are substrate for production of eicosanoids such as prostaglandins, tromboxanes, and leukotrienes. Eicosanoids which are produced from *n*-3 PUFAs were shown to have anti-inflammatory and antithrombotic effects, in contrast to those derived from *n*-6 PUFAs. Most studies concur that a decreased *n*-3 to *n*-6 PUFA ratio is linked to a higher risk of CVD, including among individuals with CKD [6,7]. In a meta-analysis by Saglimbene et al., *n*-3 PUFA supplementation was shown to reduce cardiovascular mortality in hemodialysis patients [8].

Data on the relationship between fatty acid profiles and the risk of CKD progression remain limited. Pooled analysis of 19 cohort studies and over 25,000 patients showed that a higher seafood *n*-3 PUFA level was associated with a modestly lower risk of incidence CKD and slower progression of CKD [9]. A systematic review from 2021 showed that *n*-3 PUFA supplementation reduced oxidative stress in CKD population [10]. Since the mechanisms underlying CVD in the CKD population remain incompletely understood and represent a significant clinical challenge, the aim of the present study was to investigate whether alterations in fatty acid profiles, assessed approximately a decade earlier, are associated with CKD progression and the incidence of cardiovascular events in patients with CKD. We hypothesize that higher serum *n*-3 PUFA content may be associated with better long-term cardiovascular and renal outcomes in patients with CKD. Due to limited number of patients, the aim was to perform an exploratory, hypothesis-generating analysis to identify potential associations warranting further investigation.

Additionally, we examined the impact of dietary patterns on achieving the composite study endpoint defined as renal replacement therapy, stroke, myocardial infarction, or death.

## 2. Materials and Methods

### 2.1. Experimental Approach to the Problem

In this study, we investigated whether baseline serum fatty acid profiles and dietary patterns were associated with long-term renal and cardiovascular outcomes in patients with CKD stages 1–5, whose fatty acid profiles had been previously examined a decade earlier. The hypothesis was that lower *n*-3 PUFA content and an unfavorable *n*-3/*n*-6 PUFA ratio would be associated with worse renal and cardiovascular outcomes. The composite study endpoint was defined as initiation of renal replacement therapy, stroke, myocardial infarction, or death during follow-up. The composite endpoint was chosen due to bidirectional relationship between CKD and CVD, as CKD is considered CVD equivalent, and patients with CKD are at high cardiovascular risk. Coexistence of CVD and CKD and their mutual interaction, whereby CVD accelerates CKD progression and CKD promotes cardiovascular damage, make strict separation of renal and cardiovascular outcomes clinically difficult.

### 2.2. Participants and Study Design

This study has a prospective cohort design. Participants were recruited from among CKD patients treated at the outpatient clinic of a major university-based hospital. Fatty acid profiles in these individuals had been previously determined over a decade earlier, and the results were published previously [2]. During the follow-up period, data on CKD progression, all-cause mortality, and the incidence of cardiovascular events (stroke and myocardial infarction) were collected. Data were extracted from institutional electronic medical records. 

The study was conducted in accordance with the Declaration of Helsinki. All study protocols were approved by the Local Bioethics Committee at the Medical University of Gdańsk (protocol no. NKEBN/614/2013–2014). The ethical approval date was 28 May 2014. Written informed consent for participation in the follow-up study was obtained from all participants prior to enrollment.

### 2.3. Inclusion and Exclusion Criteria

Inclusion criteria were: age ≥ 18 years, a confirmed diagnosis of CKD, and consent to participate in the study. Patients were excluded in cases of loss to follow-up.

### 2.4. Sample Selection and Follow-Up

**Figure 1:**
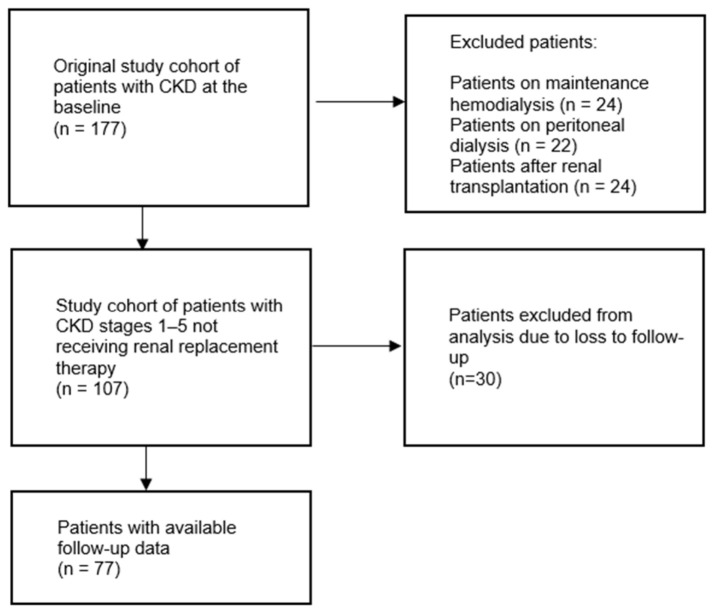
Flowchart of patient selection in the study.

A decade earlier, we evaluated the fatty acid profiles in 177 patients with CKD, including those on dialysis and after kidney transplantation. Of the original 107 participants with CKD not receiving renal replacement therapy, follow-up data were obtained for 77 patients with CKD stages 1–5. A detailed sample selection process is presented in the flowchart (Figure 1). 

### 2.5. Dietary Assessment

At the baseline, dietary habits were assessed using the FFQ-6, a validated food frequency questionnaire developed for the Polish population [11]. Data from FFQ-6 were transformed to numbers in accordance with the FFQ-6’s instructions. Associations between baseline dietary patterns and the composite study endpoint outcomes were assessed.

### 2.6. Fatty Acid Analysis

Baseline fatty acid profiles were determined at study entry. Detailed procedures for fatty acid extraction and analysis using gas chromatography–mass spectrometry (GC–MS) have been described previously [2]. Fatty acid composition was expressed as the percentage by weight of total fatty acids.

### 2.7. Outcome Definitions

The composite study endpoint was defined as initiation of renal replacement therapy, stroke, myocardial infarction, or death during follow-up. Associations between baseline dietary patterns and these outcomes were also assessed.

### 2.8. Statistical Analysis

Statistical analyses were performed using Stata/BE version 19 (StataCorp LLC, College Station, TX, USA). Data distribution was assessed using the Shapiro–Wilk test. Continuous variables are presented as mean ± standard deviation (SD) or median with interquartile range (IQR), as appropriate based on data distribution. Categorical variables are expressed as absolute frequencies and percentages. Group comparisons were conducted using the Mann–Whitney U test (Wilcoxon rank-sum test) for continuous variables and the Chi-square test for categorical variables, as appropriate. Event-free survival was estimated using the Kaplan–Meier method. Survival curves were constructed to assess differences in event-free survival between groups defined by fatty acid content, categorized according to median values. Differences between groups were evaluated using the log-rank test. Associations between fatty acid levels and the risk of the composite endpoint were assessed using Cox proportional hazards regression models. Univariable analyses were initially performed for each fatty acid, followed by multivariable models adjusted for clinically relevant covariates, including age, baseline renal function (estimated glomerular filtration rate, eGFR), and diabetes mellitus (DM). The proportional hazards assumption was formally assessed using Schoenfeld residuals and the global proportionality test. Given the limited number of events, the number of variables included in multivariable models was restricted to minimize the risk of overfitting. Hazard ratios (HRs) with 95% confidence intervals (CIs) were reported. All statistical tests were two-sided, and a *p*-value < 0.05 was considered statistically significant.

## 3. Results

### 3.1. Baseline Characteristics

Baseline clinical and biochemical characteristics of the study participants are shown in Table 1. The median age was 67.4 years and 38% were men. The median levels of SFA, MUFA, *n*-3 PUFA, *n*-6 PUFA were 33.8%, 30.7%, 2.40%, and 30.6%, respectively. During a 10-year follow-up period, five patients died, 23 required renal replacement therapy, six developed stroke, and nine experienced myocardial infarctions. Overall, 34 patients reached the composite endpoint (stroke, myocardial infarction, death, or renal replacement therapy).

### 3.2. Association Between Fatty Acid Profiles and the Composite Endpoint

**Figure 2:**
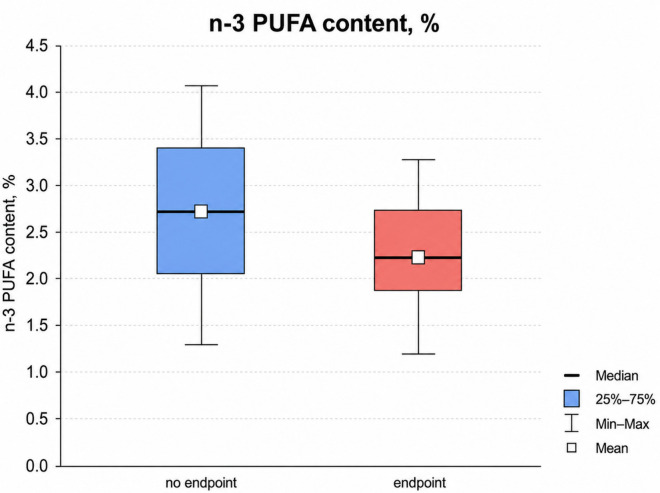
*n*-3 polyunsaturated fatty acid (PUFA) content according to composite endpoint (renal replacement therapy, myocardial infarction, stroke, or death).

Patients who reached the composite endpoint (initiation of renal replacement therapy, myocardial infarction, stroke, or death) had lower baseline *n*-3 PUFA content (2.34% [1.89–2.76] vs. 2.75% [2.16–3.41]; Mann–Whitney U test, *p* = 0.046) (Figure 2).

The *n*-3/*n*-6 ratio was also lower in patients who reached the composite endpoint albeit without reaching statistical significance (0.09 [0.07–0.11] and 0.08 [0.06–0.09], *p* = 0.068]). No significant violations of proportional hazards assumptions were identified in Cox regression analyses (all Schoenfeld residual tests *p* > 0.05). Also, no statistically significant differences in fatty acid profiles were observed in SFA, MUFA, and *n*-6 PUFA contents (Table 2).

In univariate Cox regression analyses, the levels of ALA, EPA, DHA, total SFA, and total *n*-6 PUFA were not associated with the occurrence of the composite endpoint. However, higher *n*-3 PUFA levels were significantly associated with a lower risk of the composite endpoint in Cox regression analysis (HR = 0.63; 95% CI: 0.40–0.99; *p* = 0.044). A trend toward a lower risk of the composite endpoint was observed with a higher *n*-3/*n*-6 ratio; however, this association did not reach statistical significance (*p* = 0.081) (Table 3).

In multivariable Cox regression analysis, adjusted for age, diabetes mellitus, and baseline renal function (eGFR), higher *n*-3 PUFA levels remained independently associated with a lower risk of the composite endpoint (HR = 0.60; 95% CI: 0.38–0.93; *p* = 0.021). Baseline eGFR remained a significant predictor across multivariable models, whereas age and diabetes mellitus did not reach statistical significance (Table 4).

### 3.3. Survival Analysis

**Figure 3:**
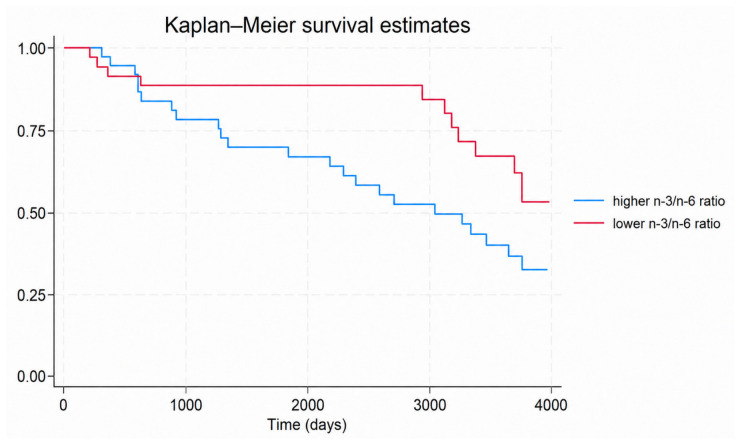
Kaplan–Meier estimates of event-free survival according to *n*-3/*n*-6 polyunsaturated fatty acids ratio (higher vs. lower).

Survival analysis was performed using the Kaplan–Meier method. Significant differences in event-free survival were observed according to the *n*-3/*n*-6 ratio with higher *n*-3/*n*-6 values associated with a more favorable prognosis (log-rank test, χ^2^ = 4.58, *p* = 0.032) (Figure 3). No statistically significant differences were found for ALA, DHA, EPA, total *n*-3 PUFA, *n*-6 PUFA, SFA, or MUFA.

### 3.4. Renal Outcomes

**Figure 4:**
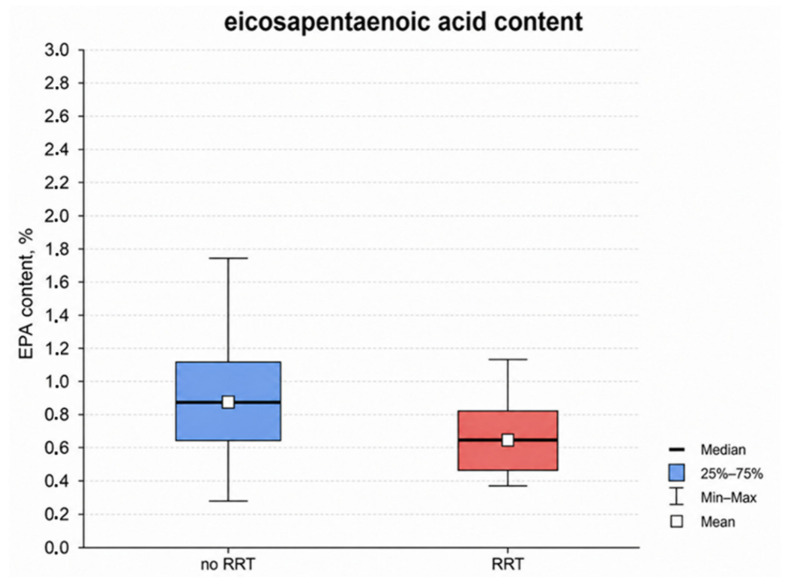
Eicosapentaenoic acid content in patients who did not require renal replacement therapy (RRT) and those who initiated RRT.

EPA content was significantly lower in patients who consequently started RRT compared with those who did not experience this outcome (median: 0.66% [0.48–0.82] vs. 0.88% [0.64–1.13], Mann–Whitney U test, *p* = 0.02) (Figure 4).

Total *n*-3 PUFA also tended to be decreased in this group of patients (2.20% [1.93–2.81] vs. 2.60% [1.93–3.06]); however, this difference did not reach statistical significance (Mann–Whitney U test, *p* = 0.29) (Table 5).

Fine–gray competing risk analysis was additionally performed to evaluate RRT initiation while accounting for death as a competing event. After adjustment for age, diabetes mellitus, and baseline eGFR, EPA was not significantly associated with RRT initiation (SHR = 1.21; 95% CI: 0.26–5.67; *p* = 0.811). Higher baseline *n*-3 PUFA levels (SHR = 0.47; 95% CI: 0.19–1.20; *p* = 0.116) and higher *n*-3/*n*-6 ratios (*p* = 0.242) demonstrated a similar protective direction of association with RRT initiation; however, statistical significance was not reached. Baseline eGFR remained independently associated with renal outcomes across all models.

Sensitivity analyses excluding patients with pre-existing cardiovascular disease at baseline were performed. Although statistical significance was attenuated after exclusion of these patients, the overall direction of associations remained similar. The reduced sample size (39 patients, 18 events) likely limited statistical power in these subgroup analyses. However, the results support the robustness of the primary findings (*p* = 0.0012).

### 3.5. Cardiovascular Outcomes

**Figure 5:**
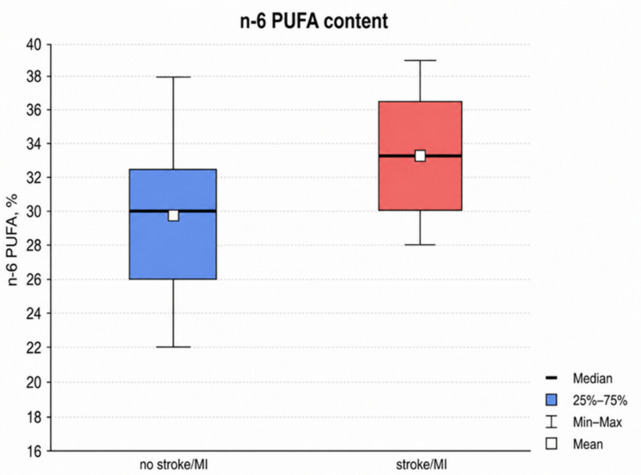
*n*-6 PUFA content in patients without events vs. myocardial infarction or stroke.

Patients who experienced stroke or myocardial infarction had significantly higher levels of *n*-6 PUFA (32.85% [29.81–36.45] vs. 29.94% [26.26–32.68], Mann–Whitney U test, *p* = 0.037) (Figure 5).

Also, patients with stroke or myocardial infarction presented with lower *n*-3/*n*-6 PUFA ratios (0.07 vs. 0.08, Mann–Whitney U test, *p* = 0.045) (Table 6). None of the individual *n*-3 fatty acids demonstrated a protective effect. There was no statistically important difference between content of total SFA, MUFA, and total *n*-3 PUFA between the two groups.

### 3.6. Association with Dietary Intake

The frequency of food intake was assessed with a food frequency questionnaire at baseline. In multivariable Cox regression analysis, no association was found between the intake of PUFA-rich foods and the occurrence of the composite endpoint, as shown in Table 7.

## 4. Discussion

CKD is an increasing global problem, including approximately 788 million patients worldwide [12]. The increasing prevalence of diabetes mellitus and the aging of the population contribute to the growing incidence CKD. More than 62% of patients undergoing dialysis are over 65 years old [13]. Reducing the risk of end-stage kidney disease remains of great importance, as it imposes a substantial burden on patients, adversely affecting quality of life, and poses significant economic challenges for society. We previously showed that patients with CKD have altered fatty acids profile and present unfavorable lower content of *n*-3 PUFA [2,3]. Lower *n*-3 PUFA content in patients with CKD may partially result from restricted diet as patients with end-stage kidney disease have been shown to have lower fish intake. This observation has been reported in both high- and low-fish-intake countries [6,14]. Other alterations in lipid profile in CKD patients include increased levels of small dense LDL particles, which are especially atherogenic, as well as high level of lipoprotein(a), which is not decreased by statin use. Reduced efficacy of statins in the CKD population may be attributable to the fact that the dyslipidemia in patients with CKD involves not only quantitative but also qualitative alterations. Therefore, there is a strong rationale for pursuing additional approaches to mitigate high CVD risk in this population. We evaluated whether a high intake of PUFA-rich foods, such as fish, nuts, and flaxseed, was associated with patient outcomes and found no statistically significant association. However, food frequency questionnaires have limited reliability, and dietary composition may have contributed to baseline differences. Its potential impact on the 10-year endpoint outcomes cannot be fully determined as baseline diet may not reflect cumulative exposure during follow-up period representing a limitation of the study.

The main finding of our follow-up study is that a lower content of *n*-3 PUFAs at baseline was significantly associated with the occurrence of the composite endpoint defined as initiation of renal replacement therapy or occurrence of stroke or myocardial infarction or death. It has been shown that *n*-3 PUFAs may modify lipid metabolism by reducing triglycerides through decreasing the hepatic synthesis of very-low-density lipoprotein cholesterol (VLDL), primarily through reduced lipogenesis and increased fatty acid β-oxidation. Other potential benefits of *n*-3 PUFAs include hypotensive, anti-thrombotic, and anti-inflammatory effects [15]. Cardiovascular risk in the CKD population is markedly exacerbated by chronic inflammation and oxidative stress, which may partially explain the protective effect associated with higher serum *n*-3 PUFA levels. These mechanisms may partially account for better outcomes in CKD patients with more favorable fatty acid profile, as shown in our study. However, it should be noted that lower *n*-3 PUFA content might be a surrogate marker of worse nutritional status, such as low albumin and cholesterol levels in patients with more advanced CKD. Therefore, an altered fatty acid profile might as well represent worse general condition, including the state of chronic inflammation.

A multivariable model restricted to clinically relevant covariates such as DM, age, and eGFR demonstrated that higher baseline *n*-3 PUFA content remained independently associated with lower risk of adverse outcomes. Nevertheless, the relatively small sample size and potential residual confounding warrant cautious interpretation.

Several studies investigated *n*-3 PUFA supplementation’s effect on cardiovascular outcome. The VITAL study on 25,871 participants, with a median follow-up of 5.3 years, showed that *n*-3 PUFA supplementation did not result in a lower incidence of major cardiovascular events, although it was associated with a significantly reduced total MI incidence [16]. This is the only large-scale randomized trial in primary prevention to this date. More studies are available in the context of secondary prevention. Earlier studies, such as the GISSI-Prevenzione and JELIS, demonstrated significant cardiovascular risk reduction [17,18]. However, as these trials were conducted using an open-label design, their findings should be interpreted with caution. A recent meta-analysis of 15 randomized controlled trials concluded that the incidence of major cardiovascular events and cardiovascular death was reduced in groups that received *n*-3 PUFA supplementation, but no differences were observed in stroke, heart failure, and all-cause mortality. Notably, the *n*-3 PUFA group exhibited an increased risk of atrial fibrillation and appeared to have a higher risk of stroke among patients with myocardial infarction. *n*-3 PUFAs were considered to have anti-arrhythmic properties, but the influence on sodium and calcium channels might also have the potential to slow down potential conduction and have pro-arrhythmic effects [19]. This observation was confirmed in a recent meta-analysis on over 114 thousand patients at high-risk of CVD in whom treatment with *n*-3-PUFAs increased the risk for atrial fibrillation [20]. In our study, the occurrence of atrial fibrillation was not evaluated, which is one of study limitations. Another important finding of our follow-up study is that *n*-6 PUFA levels were not associated with the composite endpoint. However, higher *n*-6 PUFA levels were observed in patients who experienced stroke or myocardial infarction during the 10-year follow-up period. Evidence regarding the role of *n*-6 PUFAs—whose principal representative is arachidonic acid (AA)—in CVD remains inconsistent. A high dietary intake of *n*-6 PUFAs has been suggested to promote inflammation [21]. In contrast, other studies have reported a cardioprotective effect of *n*-6 PUFA [22]. Event-free survival for the composite endpoint, assessed using the Kaplan–Meier method, differed significantly according to the *n*-3/*n*-6 PUFA ratio, with higher values associated with a more favorable prognosis. *n*-3/*n*-6 PUFA ratio seems to be of particular importance, as both classes of fatty acids compete for the same enzymatic pathways involved in eicosanoid synthesis [23].

It has been proposed that *n*-3 PUFA supplementation might decrease the risk of progression to end-stage kidney disease, mostly due to its beneficial effect on endothelium, blood pressure, oxidative stress. In a large prospective study by Koh et al., individuals without CKD with higher PUFA plasma levels were less like to develop CKD. In individuals with CKD, only DHA among PUFA was associated with a lower risk of progression of kidney failure [24]. Also, in a study by Lauretani et al., elderly patients with higher plasma PUFAs had a lower risk of developing renal insufficiency. High concentrations of both *n*-3 PUFAs and *n*-6 PUFAs attenuated the decline in renal function among older patients [25]. In a cross-sectional study by Higashiyama, the content of *n*-3 PUFAs in the serum was positively associated with eGFR. Our study yielded similar results, the median of ALA, DHA, and EPA contents, as well as the total *n*-3 PUFA, were lower in patients who required renal replacement therapy during the follow-up period, but this was only statistically significant for EPA. In a meta-analysis by Hu et al., *n*-3-PUFA supplementation was associated with a reduced risk of end-stage kidney disease [26]. There have also been studies suggesting that *n*-3 PUFAs might decrease proteinuria in CKD patients [27]; however, these nephroprotective effects have not been confirmed in further studies [28].

Another limitation of the present study is a concern that a single baseline fatty acid measurement might not fully reflect the long-term fatty acid profile during follow-up. Previous studies have shown that fatty acid profiles may change over time [29]. However, Djoussé et al. demonstrated a correlation between plasma phospholipid fatty acids spaced apart by 15 years, and concluded that a single measurement might be reasonable to estimate the risk of heart failure over long-term follow up [30]. Other study limitations include relatively small sample size and limited statistical power. Therefore, presented findings should be regarded as exploratory and hypothesis-generating. Further studies in larger, independent cohorts are needed to validate these preliminary observations. Confounding factors related to pharmacological treatment or supplementation which were not assessed also cannot be excluded. Another limitation is the absence of data on the incidence of atrial fibrillation, which has become an increasingly important clinical concern. A major strength of the present study is the long follow-up period of 10 years.

## 5. Conclusions

In conclusion, our findings demonstrate that lower serum *n*-3 PUFA levels were associated with adverse long-term outcomes, including stroke, myocardial infarction, initiation of renal replacement therapy, and death among patients with CKD. Moreover, a higher *n*-3/*n*-6 PUFA ratio conferred a cardioprotective effect in survival analysis. However, it has to be taken into account that a higher level of serum *n*-3 PUFA may be a surrogate marker of a better nutritional status and healthier lifestyle, which might decrease the risk of hypertension, obesity, and diabetes as acknowledged risk factors for developing CKD.

Given the opposing roles of *n*-6 and *n*-3 PUFAs in the generation of pro- and anti-inflammatory eicosanoids, maintaining an adequate *n*-3/*n*-6 PUFA ratio may be of particular importance in this population. Our findings confirm that a more favorable fatty acid profile may contribute to improved cardiovascular and renal outcomes in CKD patients. However, due to small number of patients and endpoint events, the results of the should be interpreted with caution and considered exploratory.

The role of fatty acid profiles in long-term outcomes among patients with CKD warrants further investigation, as CVD remains the leading cause of death in this population. Further studies with larger cohorts, ideally including clinical trials, are needed to determine whether modifying fatty acid profiles can causally influence renal and cardiovascular outcomes.

## Figures and Tables

**Table 1:** Study group characteristics.

Number of participants, *n*	77
Men, *n* (%)	29 (37.66)
Age, years, median (IQR)	67.4 (59.0–72.0)
BMI, kg/m^2^, mean (SD)	29.55 (5.26)
DM, *n*, %	30 (38.96)
CVD, *n* %	37 (48.05)
Hypertension, *n* (%)	73 (94.81)
Hemoglobin, g/dL, mean (SD)	13.28 (1.91)
CRP, mg/L, median (IQR)	2.12 (2.03–5.60)
Creatinine, mg/dL, median (IQR)	1.7 (1.38–2.41)
BUN, mg/dL, median (IQR)	29.9 (23.2–42.8)
Glucose mg/dL, median (IQR)	103 (95.5–133.0)
HOMA-IR, median (IQR)	3.55 (2.25–5.70)
Albumin g/L, median (IQR)	38.0 (36.0–40.0)
Total cholesterol mg/dL, mean (SD)	198.99 (52.55)
Triglycerides mg/dL, mean (SD)	150.19 (58.32)
HDL, mg/dL, median (IQR)	43.0 (38.0–55.0)
LDL, mg/dL, mean (SD)	122.39 (45.80)
*n*-3 PUFA, %, median (IQR)	2.40 (1.93–3.05)
EPA, %, median (IQR)	0.86 (0.60–1.06)
DHA, %, median (IQR)	1.00 (0.84–1.31)
ALA, %, median (IQR)	0.26 (0.18–0.32)
*n*-6 PUFA, %, median (IQR)	30.64 (27.09–33.05)
AA, %, median (IQR)	5.04 (4.59–5.75)
MUFA, %, median (IQR)	30.74 (28.05–33.30)
SFA, %, median (IQR)	33.82 (32.38–34.88)

Abbreviations: IQR, interquartile range; BMI, body mass index; SD, standard deviation; DM, diabetes mellitus; CVD, cardiovascular disease; CRP, C-reactive protein; BUN, blood urea nitrogen; HOMA-IR, homeostatic model assessment of insulin resistance; HDL, high-density lipoprotein cholesterol; LDL, low-density lipoprotein cholesterol; PUFA, polyunsaturated fatty acid; *n*-3, omega-3 fatty acid; *n*-6, omega-6 fatty acid; EPA, eicosapentaenoic acid; DHA, docosahexaenoic acid; ALA, alpha-linolenic acid; AA, arachidonic acid; MUFA, monounsaturated fatty acid; SFA, saturated fatty acid.

**Table 2:** Fatty acid profiles according to composite endpoint (RRT, myocardial infarction, stroke, or death).

	No Endpoint Median (IQR)	Composite Endpoint Median (IQR)	*p*-Value
ALA	0.26 (0.18–0.32)	0.26 (0.18–0.39)	0.649
EPA	0.74 (0.61–1.08)	0.75 (0.59–1.02)	0.748
DHA	1.00 (0.77–1.33)	1.03 (0.91–1.31)	0.823
SFA	33.83 (31.83–34.88)	33.71 (32.06–34.70)	0.497
MUFA	30.74 (28.41–33.42)	30.71 (27.06–32.74)	0.631
*n*-3 PUFA	2.62 (2.16–3.41)	2.24 (1.89–2.76)	0.048 *
*n*-6 PUFA	29.77 (27.09–33.06)	30.88 (27.39–32.88)	0.721
*n*-3/*n*-6 ratio	0.089 (0.068–0.106)	0.077 (0.059–0.091)	0.068

Values are presented as median and interquartile range (IQR). * *p* < 0.05 (Mann–Whitney U test). Abbreviations: IQR, interquartile range; ALA, alpha-linolenic acid; EPA, eicosapentaenoic acid; DHA, docosahexaenoic acid; SFA, saturated fatty acid; MUFA, monounsaturated fatty acid; PUFA, polyunsaturated fatty acid.

**Table 3:** Univariate Cox proportional hazards analysis for the composite endpoint.

	HR (95% CI)	*p*-Value
ALA	2.70 (0.09–80.55)	0.567
DHA	0.82 (0.34–1.99)	0.662
EPA	0.86 (0.33–2.24)	0.750
SFA	1.12 (0.97–1.31)	0.124
MUFA	0.99 (0.90–1.08)	0.742
*n*-3 PUFA	0.63 (0.40–0.99)	0.044
*n*-6 PUFA	1.00 (0.92–1.08)	0.921
*n*-3/*n*-6 PUFA ratio	0.00002 (0.00–3.66)	0.081

Abbreviations: HR, hazard ratio; CI, confidence interval; ALA, alpha-linolenic acid; DHA, docosahexaenoic acid; EPA, eicosapentaenoic acid; SFA, saturated fatty acid; MUFA, monounsaturated fatty acid; PUFA, polyunsaturated fatty acid.

**Table 4:** Multivariate Cox proportional hazards analysis for the composite endpoint.

	HR	Std. Error	z	*p*-Value	95% CI (Lower–Upper)
*n*-3 PUFA	0.60	0.13	−2.30	0.021	0.38–0.93
Age	0.98	0.01	−1.59	0.111	0.95–1.00
DM	1.59	0.56	1.32	0.188	0.80–3.17
eGFR	0.94	0.01	−4.39	0.000	0.91–0.97

Abbreviations: HR, hazard ratio; CI, confidence interval; PUFA, polyunsaturated fatty acid; DM, diabetes mellitus; eGFR, estimated glomerular filtration rate.

**Table 5:** Fatty acid profiles in patients who did not require RRT and those who initiated RRT.

	No RRT Median (IQR)	RRT Median (IQR)	*p*-Value
ALA	0.27 (0.20–0.33)	0.22 (0.15–0.33)	0.084
EPA	0.88 (0.64–1.13)	0.66 (0.48–0.82)	0.020 *
DHA	1.06 (0.84–1.33)	0.97 (0.73–1.27)	0.293
Total SFA	33.57 (31.96–34.73)	34.12 (32.53–35.48)	0.423
MUFA	30.26 (28.02–33.32)	31.06 (29.35–33.02)	0.522
*n*-3 PUFA	2.60 (1.93–3.06)	2.20 (1.93–2.81)	0.293
*n*-6 PUFA	30.94 (27.82–33.19)	30.24 (25.84–32.68)	0.312
*n*-3/*n*-6 ratio	0.08 (0.06–0.10)	0.08 (0.06–0.10)	0.718

Values are presented as median and interquartile range (IQR). * *p* < 0.05. Abbreviations: RRT, renal replacement therapy; ALA, alpha-linolenic acid; EPA, eicosapentaenoic acid; DHA, docosahexaenoic acid; SFA, saturated fatty acid; MUFA, monounsaturated fatty acid; PUFA, polyunsaturated fatty acid.

**Table 6:** Fatty acid profiles in patients without events vs. myocardial infarction or stroke.

	No Event Median (IQR)	MI/Stroke Median (IQR)	*p*-Value
Total SFA	33.83 (32.16–34.88)	33.69 (32.65–34.97)	0.978
MUFA	31.06 (28.56–33.42)	27.95 (26.35–31.87)	0.063
*n*-3 PUFA	2.53 (1.93–3.08)	2.25 (1.62–2.68)	0.171
*n*-6 PUFA	29.94 (26.26–32.68)	32.85 (29.81–36.45)	0.037 *
*n*-3/*n*-6 ratio	0.08 (0.06–0.10)	0.07 (0.05–0.08)	0.045 *

Values are presented as median and interquartile range (IQR). * *p* < 0.05 (Mann–Whitney U test). Abbreviations: IQR, interquartile range; MI, myocardial infarction; SFA, saturated fatty acid; MUFA, monounsaturated fatty acid; PUFA, polyunsaturated fatty acid.

**Table 7:** Association between selected food intake and the composite endpoint.

Food Group	HR (95% CI)	*p*-Value
Lean fish (e.g., cod, pollock)	1.56 (0.86–2.85)	0.146
Fatty fish (e.g., salmon, sardines)	1.19 (0.75–1.88)	0.464
Nuts (e.g., peanuts, hazelnuts, walnuts)	0.92 (0.64–1.33)	0.662
Seeds (e.g., pumpkin, sesame, sunflower)	0.85 (0.58–1.25)	0.415

HR—hazard ratio; CI—confidence interval. Results from Cox regression analysis for the composite endpoint.

## Data Availability

The datasets used during the current study are available from the corresponding author upon reasonable request. The data are not publicly available due to privacy.

## Abbreviations

The following abbreviations are used in this manuscript:

CVD	Cardiovascular disease
CKD	Chronic kidney disease
PUFA	Polyunsaturated fatty acid
SFA	Saturated fatty acid
MUFA	Monounsaturated fatty acid
EPA	Eicosapentaenoic acid
DHA	Docosahexaenoic acid
FFQ	Food frequency questionnaire
GC-MS	Gas chromatography–mass spectrometry
SD	Standard deviation
IQR	Interquartile range
eGFR	Estimated glomerular filtration rate
HR	Hazard ratio
CI	Confidence interval
BMI	Body mass index
DM	Diabetes mellitus
CRP	C-reactive protein
BUN	Blood urea nitrogen
HOMA-IR	Homeostatic model assessment of insulin resistance
HDL	High-density lipoprotein cholesterol
LDL	Low-density lipoprotein cholesterol
ALA	Alpha-linolenic acid
AA	Arachidonic acid
RRT	Renal replacement therapy
